# Response of Canopy Photosynthesis, Grain Quality, and Harvest Index of Wheat to Different Nitrogen Application Methods

**DOI:** 10.3390/plants11182328

**Published:** 2022-09-06

**Authors:** Xiangqian Zhang, Shizhou Du, Yunji Xu, Yuqiang Qiao, Chengfu Cao, Wei Li

**Affiliations:** 1 Crops Research Institute, Anhui Academy of Agricultural Sciences, Crop Building, No. 40, Nongke South Road, Luyang District, Hefei 230031, China; 2 Joint International Research Laboratory of Agriculture and Agri-Product Safety, The Ministry of Education of China, Yangzhou University, Yangzhou 225009, China

**Keywords:** root traits, chlorophyll content, photosynthetic rate, grain quality, N fertilization, wheat

## Abstract

To fully explore the effects of N on enhancing photosynthesis, grain quality, and yield of wheat (Ningmai 13), experiments with four nitrogen levels 0 (N0), 120 (N1), 180 (N2), and 240 (N3) kg N ha^−^^1^ and four ratios of basal to topdressing R0 (0:0), R1 (7:3), R2 (6:4), and R3 (5:5) were conducted. The basal N was applied to soil before sowing and the topdressing N was applied at jointing stage. The effect of N topdressing ratio on improving leaf area of photosynthetic efficiency was insignificant under the same N level. The effect of N fertilization level on increasing chlorophyll content was more significant than that of N topdressing ratio. Within the same N level, the canopy photosynthetically active radiation in R2 was higher than that in R1 and R3, and increasing N by 60 kg ha^−1^ significantly enhanced canopy photosynthetically active radiation. The effect of N topdressing ratio on photosynthetic rate, stomatal conductance, and transpiration rate were consistently R2 > R3 > R1; compared to N1, N3 could significantly increase photosynthetic rate. Increasing 120 kg N ha^−1^ significantly enhanced grain protein content, wet gluten, and sedimentation value, while the effect of N topdressing ratio was insignificant. Increasing N dose from 120 kg ha^−1^ to 180 kg ha^−1^ significantly enhanced yield, and the yields and harvest indexes in 2019, 2020, and 2021 were consistently R2 > R3 > R1. The findings suggested that the effect of increasing N dose (60 kg ha^−1^) was more considerable than that of N topdressing ratio, N3R2 (within the range of N application in this experiment) was more conducive to improving canopy photosynthesis, yield, and harvest index, and R3 was more conducive to increasing grain protein content, wet gluten, and sedimentation value.

## 1. Introduction

In recent years, farmers have increased the application dose of nitrogen fertilizer in crop production in order to improve yield, grain quality, and achieve more economic profit. Although increasing N application is an important practice for agricultural production, crop yield, economic profit, and N application rates are not always positively correlated [1]. For example, China has about 10% of the world’s arable land, but has consumed about 40% of global N fertilizer, resulting in relatively low crop yield increases (only 22%) and the largest N loss. Also, ref. [2] has reported that overuse and mismanagement of N fertilizer could lead to higher tolerance to N fertilizers in cultivars, resulting in a decrease in the effect of N fertilizer on improving crop growth and development, grain quality, harvest index, and increasing yield. How to determine the appropriate N application dose and method may mainly depend on whether the leaf area, photosynthesis, grain quality, yield, and harvest index of crops can be effectively improved. 

Photosynthesis is the fundamental basis of crop growth and development, and the improvement of photosynthetic efficiency can therefore enhance crop yields. The N in soil and crop plants is an important element influencing photosynthesis in agricultural production, and the change of nitrogen application rate and period and N topdressing ratio usually leads to the increase or decrease in N in soil and plants. The method of nitrogen application can affect the growth and development, yield, and harvest index of crops, mainly because the existing state of N in soil and plant has a very close relationship with the leaf area index (LAI), chlorophyll content, photosynthetic rates, radiation interception, and radiation-use efficiency of crops [3,4,5]. Studies have shown that intercellular CO_2_ concentration, stomatal conductance, chlorophyll content, leaf area, photosynthetically active radiation (PAR), and net photosynthetic rates decreased or increased with changes in nitrogen application rate and N topdressing ratio [6,7,8]. Accordingly, increasing photosynthetic capacity and efficiency by improving nitrogen application methods is an important way for raising potential crop yields and harvest index.

Among agronomic techniques, N fertilization is largely regarded as the main factor affecting nutritional quality and processing quality of grain. Nitrogen fertilization usually contributes to an increase in protein content, especially when fertilizer rates satisfy the requirements of both protein and yield synthesis [9]. Several studies have reported that application of N in postanthesis can directly increase grain protein content without reducing wheat yield [10,11]. Ref. [12] not only found that applying half of the recommended N rate (120 kg ha^−1^) at planting and the rest at tillering resulted in a high total yield, high grain N uptake, and the highest GPC (grain protein content) price premium, but also reported that split application of N fertilizer could help to synchronize N supply with wheat N demand and reduce N loss. Therefore, to optimize nitrogen fertilization in wheat production, it is necessary to have a better understanding of the wheat yield and grain quality in response to different nitrogen fertilization methods. 

Wheat is an important food crop and a relatively high N-consuming crop, and wheat grain yield is closely related to the N fertilizer application rate and method. In order to enhance wheat yield, appropriate N application techniques should be studied and adopted. Research has proven that appropriate synchronization between the rate and timing of N application greatly helps to improve crop growth, photosynthesis, yield, grain quality, and nitrogen use efficiency [13,14]. Split application of urea, where a portion is applied before seeding and a portion is applied at later growth stages, may be an effective way to increase wheat yield. In China, about 70~100% of N fertilizer is used as basal fertilizer, but too much N used as basal fertilizer may be a major cause of the waste of fertilizer due to the asynchrony between N supply and N requirements. Understanding leaf area, canopy photosynthesis, grain quality, yield, and harvest index in response to different N application dose and N topdressing ratio has great significance on optimizing N efficiency and economic benefit of nitrogen fertilizer. 

## 2. Result

### 2.1. Effects of Nitrogen Application Method on Photosynthesis 

#### 2.1.1. Leaf Area of Photosynthetic Efficiency

The areas of the flag leaf (Table 1), the second leaf, and the third leaf from the top in N0 were significantly lower than that in N1, N2, and N3. Within the same N level (N1, N2, or N3), the areas of the flag leaf, the second leaf, and the third leaf from top in R2 were slightly higher than that in R1 and R3, but without significant differences among R1, R2, and R3, indicating that the effect of ratio of basal to topdressing on improving leaf area of photosynthetic efficiency was insignificant under the same N level. Within the same ratio of basal to topdressing (R1, R2, or R3), increasing N applications enhanced the area of the flag leaf, the second leaf, and the third leaf from top, and the leaf area of photosynthetic efficiency in N3 was significantly higher than that in N1. Compared to N1, N3 for R2 significantly increased the area of flag leaf, the second and the third leaf from top by 19.57%, 12.94%, 8.78% in 2020 and 18.37%, 8.50%, 8.24% in 2021, respectively. Within the same ratio of basal to topdressing, increasing N by 120 kg ha^−1^ (N3 compared to N1, N1 compared to N0) significantly enhanced leaf area of photosynthetic efficiency. 

#### 2.1.2. Chlorophyll Content 

**Figure 1:**
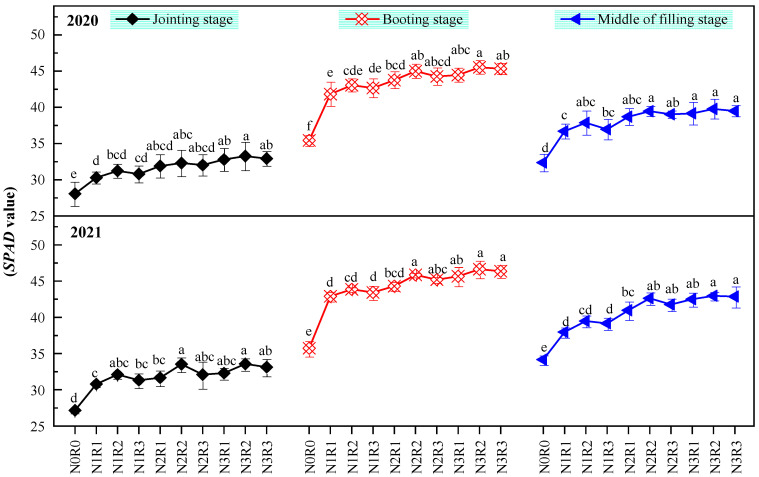
The effects of nitrogen application method on chlorophyll content of flag leaf. Values were means ± SD. Different letters indicated significant difference within the same year under the treatments of four nitrogen levels and four ratios of basal to topdressing by LSD test (ANOVA) at the 5% level. Four nitrogen fertilization levels: N0, 0 kg ha^−1^; N1, 120 kg ha^−1^; N2, 180 kg ha^−1^; N3, 240 kg ha^−1^. Four ratios of basal N to topdressing: R0, 0:0; R1, 7:3; R2, 6:4; R3, 5:5.

The chlorophyll contents (Figure 1) at each growth stage in N0 were significantly lower than that in N1, N2, and N3. Within the same N level (N1, N2, or N3), the difference in chlorophyll content at each growth stage among R1, R2, and R3 in 2020 and 2021 (except for under N2) were insignificant. Within the same N level, the chlorophyll content in R2 was higher than that in R1 and R3. Within the same ratio of basal to topdressing (R1, R2, or R3), the chlorophyll content in N3 was significantly higher than that in N1 at jointing and booting stages in 2020 and at booting and middle of filling stages in 2021, respectively. The difference in chlorophyll content between N3 and N2 was insignificant (within the same ratio of basal to topdressing). Compared to N1, N3 for R1 and R3 significantly increased chlorophyll content by 8.25%, 6.46%, 6.73% and 6.82%, 6.09%, 6.84% at jointing, booting and middle of filling stages in 2020, respectively. The results showed that the effect of N fertilization level was more significant than the ratio of basal to topdressing on increasing chlorophyll content. 

#### 2.1.3. Canopy Photosynthetically Active Radiation 

**Figure 2:**
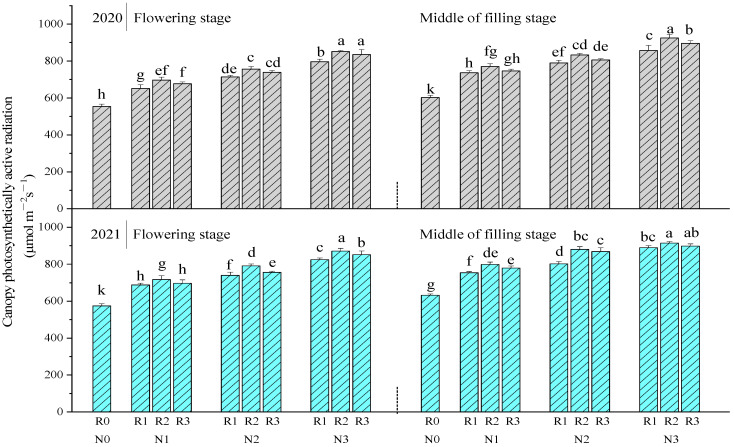
The effects of nitrogen application method on canopy photosynthetically active radiation. Values were means ± SD. Different letters above columns indicated significant difference within the same year under the treatments of four nitrogen levels and four ratios of basal to topdressing by LSD test (ANOVA) at the 5% level. Four nitrogen fertilization levels: N0, 0 kg ha^−1^; N1, 120 kg ha^−1^; N2, 180 kg ha^−1^; N3, 240 kg ha^−1^. Four ratios of basal N to topdressing: R0, 0:0; R1, 7:3; R2, 6:4; R3, 5:5.

The canopy photosynthetically active radiation (Figure 2) in N0 was significantly lower than that in N1, N2, and N3. Within the same N level (N1, N2, or N3), the canopy photosynthetically active radiation in R2 was higher than that in R1 and R3. Compared to R1, R2 for N1 significantly increased canopy photosynthetically active radiation by 6.96% and 4.59% at flowering and middle of filling stages in 2020, and significantly increased by 4.28% and 5.98% in 2021, respectively. Within the same ratio of basal to topdressing (R1, R2 or R3), the canopy photosynthetically active radiation in N2 was significantly higher than that in N1, and in N3 was significantly higher than that in N2. When compared to N2, N3 for R2 significantly increased canopy photosynthetically active radiation by 12.53% and 11.11% at flowering and middle of filling stages in 2020, and by 10.17% and 3.90% in 2021, respectively. The results showed that increasing N by 60 kg ha^−1^ (N2 compared to N1, N3 compared to N2) could significantly enhance the canopy photosynthetically active radiation under the same ratio of basal to topdressing. 

#### 2.1.4. Photosynthetic Characteristics

As shown in Table 2, within the same N level (N1, N2, or N3), the effect of the ratio of basal to topdressing on photosynthetic rate, stomatal conductance, and transpiration rate were consistently R2 > R3 > R1 in 2020 and 2021, but the differences among R1, R2, and R3 were insignificant. The value of intercellular CO_2_ concentration was the highest in R1, and the lowest in R2, but the differences among R1, R2, and R3 were insignificant. Within the same ratio of basal to topdressing (R1, R2 or R3), the photosynthetic rate, stomatal conductance, and transpiration rate in N3 were significantly higher than those of N1 (except for under R1 in 2020); when compared to N1, N3 for R1, R2, R3 significantly increased photosynthetic rate by 15.63%, 18.74%, 18.14% in 2020 and 32.12%, 24.33%, 29.39% in 2021, respectively. The intercellular CO_2_ concentration in N1 was significantly higher than that in N3 under the same ratio of basal to topdressing. The results showed that the photosynthetic rate was not significantly affected by the ratio of basal to topdressing under the same N level and that increasing N by 120 kg ha^−1^ significantly enhanced photosynthetic rate of wheat. 

### 2.2. Effects of Nitrogen Application Method on Grain Quality 

Increasing N application dose (Table 3) enhanced the content of protein, wet gluten, kernel hardness, and sedimentation value, while decreased the percentage content of starch. Within the same N level (N1, N2, or N3), the protein content, wet gluten, kernel hardness, and sedimentation value were the highest in R3 and the lowest in R1, while the starch content was the highest in R1 and the lowest in R3. The differences in protein content, starch content, wet gluten, kernel hardness, and sedimentation value among R1, R2, and R3 were insignificant under the same N level. Within the same ratio of basal to topdressing (R1, R2, or R3), the protein content, wet gluten, and sedimentation value in N3 were significantly higher than that in N1, while the starch content was significantly lower than that of N1, the difference in sedimentation value between N2 and N1 was significant, while the difference in kernel hardness between N2 and N1 was insignificant. Compared to N1, N3 for R1 and R3 increased protein content, wet gluten, kernel hardness, sedimentation value by 9.57%, 10.81%, 18.60%, 3.00% and 10.32%, 11.22%, 18.06%, 3.64%, respectively. The results revealed that the effect of the ratio of basal to topdressing on improving grain quality indexes was insignificant, increasing N by 120 kg ha^−1^ (N1 compared to N0, N3 compared to N1) significantly enhanced grain protein content, wet gluten, and sedimentation value. 

### 2.3. Effects of Nitrogen Application Method on Yield and Harvest Index

**Figure 3:**
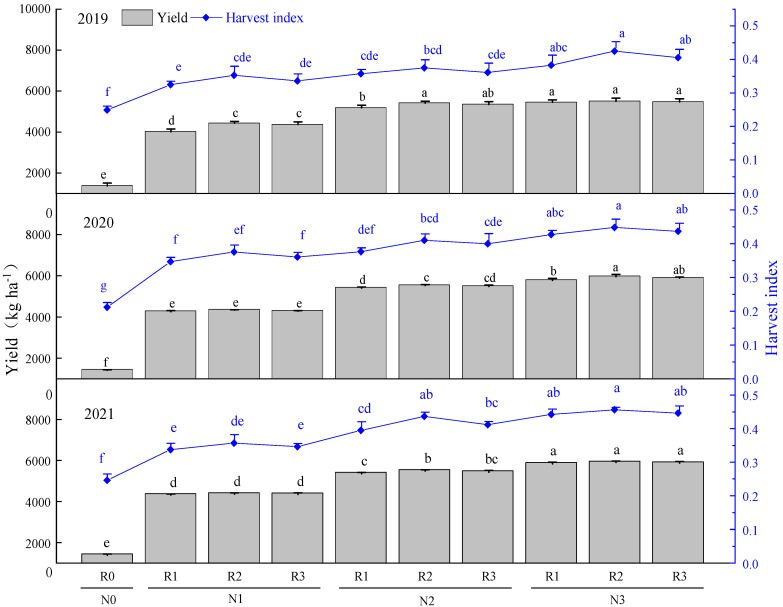
The effects of nitrogen application method on yield and harvest index. Values were means ± SD. Different letters above columns indicated significant difference within the same year under the treatments of four nitrogen levels and four ratios of basal to topdressing by LSD test (ANOVA) at the 5% level. Four nitrogen fertilization levels: N0, 0 kg ha^−1^; N1, 120 kg ha^−1^; N2, 180 kg ha^−1^; N3, 240 kg ha^−1^. Four ratios of basal N to topdressing: R0, 0:0; R1, 7:3; R2, 6:4; R3, 5:5.

As shown in Figure 3, the yield and harvest index (2019, 2020, and 2021) in N0 were significantly lower than that in other nitrogen fertilization treatments. Within the same ratio of basal to topdressing (R1, R2, or R3), the yield in N2 and N3 was significantly higher than that in N1, indicating that increasing N application dose from 120 kg ha^−1^ to 180 kg ha^−1^ significantly enhanced yield. Within the same N level, the yields of three years were the highest in R2, and the lowest in R1, and the differences between R2 and R3 were insignificant. Within the same ratio of basal to topdressing, the harvest indexes in 2019, 2020, and 2021 were consistently N3 > N2 > N1, and the difference between N3 and N1 was significant. Within the same N level (N1, N2, or N3), compared to R1 and R3, R2 enhanced harvest index, and the differences between R1, R2 and R3 were insignificant (except between R1 and R2 under N2 in 2021). The results revealed that increasing N application dose appropriately with the ratio of basal to topdressing R2 was relatively more conducive to improving yield and harvest index (within the range of N application in this experiment).

### 2.4. Correlation Analysis

**Figure 4:**
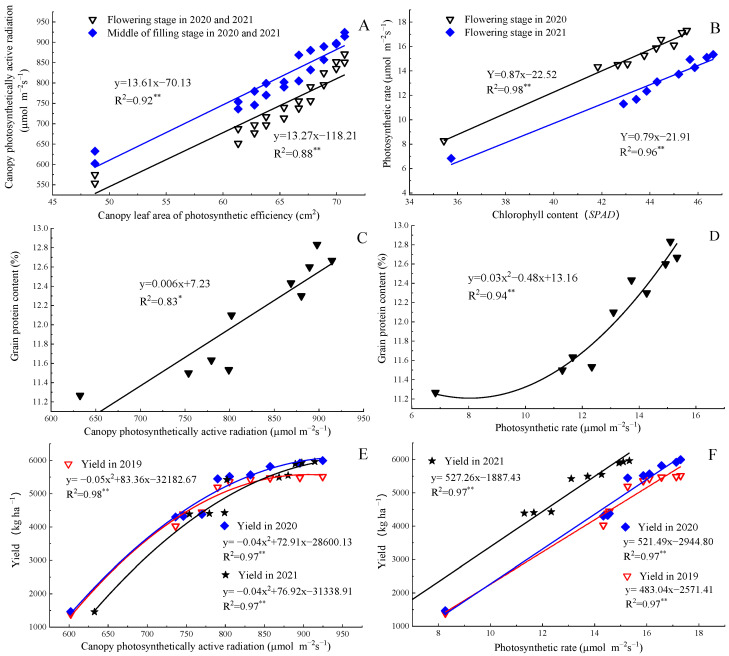
Relationship between canopy leaf area of photosynthetic efficiency and canopy photosynthetically active radiation (**A**), chlorophyll content and photosynthetic rate (**B**), canopy photosynthetically active radiation and grain protein content (**C**), photosynthetic rate and grain protein content (**D**), canopy photosynthetically active radiation and yield (**E**), photosynthetic rate and yield (**F**). * Correlation is significant (*p* < 0.05); ** Correlation is markedly significant (*p* < 0.01).

Figure 4 showed that the canopy leaf area of photosynthetic efficiency was significantly positively correlated to canopy photosynthetically active radiation with the shape of a straight line in 2020 and 2021. The chlorophyll content had a markedly significant relationship with photosynthetic rate. The canopy photosynthetically active radiation was significantly positively correlated to grain protein content with the shape of a straight line, and the photosynthetic rate was significantly positively correlated to grain protein content with the shape of a quadratic polynomial curve. In 2019, 2020, and 2021, the results showed that the relationships between canopy photosynthetically active radiation and yield were quadratic parabolas, the relationships between photosynthetic rate and yield were straight lines, and the coefficients of determination (*R*^2^) were all above 0.95, indicating that increasing photosynthetic rate could effectively improve crop yield, but the yield would no longer considerably increase or even decrease when canopy photosynthetically active radiation increased to a certain extent (this may be due to high population density of plants).

## 3. Discussion

Photosynthesis is necessary for crop growth and development, and chlorophyll, leaf area, canopy photosynthetically active radiation, and photosynthetic rate play an extremely important role in the absorption and utilization of light energy. The authors of [15] found that nitrogen deficiency (low N application) significantly reduced leaf area, leaf chlorophyll content, and photosynthetic rate, resulting in lower biomass production; [16] indicated that N topdressing increased foliar N concentration and photosynthetic rate when compared to the treatment of without N topdressing. Also, [17] reported that increasing N application rate enhanced the leaf area index, chlorophyll content, and photosynthesis of crops, and compared to CK (0 kg N ha^−1^) and 100 kg N ha^−1^, 250 kg N ha^−1^ showed the best effect on photosynthesis. In this study, we observed that within the same N level, R2 was more conducive to increasing photosynthetic efficiency leaf area of canopy, chlorophyll content, canopy photosynthetically active radiation, and photosynthetic rate; the photosynthetic indexes could be considerably improved by increasing N application dose. In addition, we also found that increasing N by 120 kg ha^−1^ significantly enhanced the canopy photosynthetically active radiation and photosynthetic rate. N application dose and topdressing ratio considerably affected photosynthetic indexes mainly because the photosynthetic physiological processes of plants can be regulated by leaf and soil N content and status, which are closely correlated with chlorophyll content, leaf area, and photosynthetic rate [18,19], and the leaf and soil N can be increased or decreased by matching N application dose, period, and N topdressing ratio with plant demand through reasonable N application methods. 

Nitrogen is widely considered as the main factor that can directly affect storage proteins, as well as the flour quality and nutritional quality of grain [9]. In terms of N application methods, such as N application dose and topdressing period and ratio, several studies have proved that delaying the N application in spring to the end of stem elongation can promote grain protein content accumulation more than yield, and dividing the total N application into two or more stages (N topdressing) seemed to have a greater influence on the grain quality than the same total N all used as basal fertilizer [20,21]. Ref. [12] investigated the effect of N application level and N topdressing ratio on grain quality and found that applying half of the recommended level (120 kg N ha^−1^) at planting and the rest at tillering resulted in a high grain N uptake and the highest grain protein content price premium. In our study, we observed that N topdressing 5:5 was more conducive to increasing protein content, wet gluten, kernel hardness, and sedimentation value; increasing N application dose enhanced protein content, wet gluten, kernel hardness, and sedimentation value, while decreasing starch content. The main reasons for increases in N application dose and topdressing ratio considerably influencing grain quality are as follows: (i) grain quality is determined by both genetic and environmental factors, and N availability or rate could have much greater impact on quality than genetic factors [22]; (ii) the regulation of soil N and artificial nitrogen application directly affects source–sink translocation and thus affects grain quality [23,24]; (iii) enhancing N topdressing ration can make crops absorb more nitrogen in the middle and late growth period (the development of crop root system is relatively perfect), and the dose of N absorbed by crops is closely related to grain quality [25]. Ref. [26] also reported that the determination of protein content in grains depends on the properties of N absorption, accumulation, and translocation in the crop plant, the effects of a genetic factor for controlling grain protein content could be altered by N application rate and topdressing ratio. 

Nitrogen application rate and topdressing ratio are the most important management factors affecting grain yield and harvest index of crops. Ref. [27] reported that the split of N fertilization and modification of topdressing timing are commonly recommended approaches in intensive wheat production for achieving satisfying yield without increasing N dose. Refs. [28,29] also found that the grain yield and harvest index might be considerably affected by nitrogen application rate and topdressing ratio. We found that the yields in 2019, 2020, and 2021 were consistently R2 > R3 > R1, and the effect of topdressing ratio R2 on improving wheat harvest index was better than that of R1 and R3. In addition, increasing the N application dose from 120 kg ha^−1^ to 180 kg ha^−1^ significantly enhanced yield under the same N topdressing ratio, and the harvest index could be improved by increasing nitrogen application dose (within the range of N application in this experiment). These results agree with [14,30], who also proved that increasing N application rate appropriately with reasonable ratio of basal to topdressing could considerably improve grain yield, and it is well-known that the increase or decrease of grain yield will cause the change of crop harvest index. N application dose and topdressing ratio can considerably affect grain yield and harvest index mainly because (i) N is the most important nutrient in terms of yield and population biomass formation [28]; (ii) wheat requires less N fertilizer at seedling stage, and its basal N fertilizer utilization efficiency is relatively low; therefore, appropriate reduction of the N application dose at the seedling stage and reasonable determination of N topdressing ratio in later growth stages may be effective for enhancing N use efficiency and yield by better matching N supply with plant demand [12,31]; (iii) in the normal range of N application, increasing N application dose and appropriate N topdressing ratio can effectively improve the root traits, green leaf area, chlorophyll content, canopy photosynthesis, growth, and development of plants, which are closely related to yield and harvest index. 

## 4. Materials and Methods

### 4.1. Experimental Design and Management 

The experiment was conducted in the Baihu farm, Lujiang County, Hefei City (31°53′ N, 117°14′ E; 29.8 m a.s.l.), Anhui Province in China from 2018 to 2021. The region is classified as having a subtropical monsoon climate. Annual mean precipitation is 1050–1250 mm, and 50% occurs from April to August. The annual mean temperature is 16.4 °C and accumulated temperatures above 10 °C were 4800–5400 °C. The frost-free period is 255–270 days each year. The basic physical and chemical properties of soil (0~20 cm) at the beginning of the nitrogen fertilization experiment was total N of 1.24 g kg^−1^, total P 0.58 g kg^−1^, available N 98.10 mg kg^−1^, available P 7.50 mg kg^−1^, organic matter 18.22 g kg^−1^, and pH 5.8. 

The experiment was conducted in a randomized block design with four N fertilization levels (N0, N1, N2, and N3) and four ratios of basal to topdressing (R0, R1, R2, and R4) as the treatment variables. The experiment included two variants (N application dose and N topdressing ratio), and the experimental plan generated 10 treatments and each treatment was replicated three times. Based on our previous study and the economic benefits of N application, we set the maximum N dose as the conventional N application dose in Chinese wheat production; then, different N topdressing ratios were adopted. The four N levels were N0 (0 kg ha^−1^), N1 (120 kg ha^−1^), N2 (180 kg ha^−1^), and N3 (240 kg ha^−1^); the four ratios of basal N to topdressing were R0 (0:0), R1 (7:3), R2 (6:4), and R3 (5:5), and the topdressing N was applied at jointing stage of wheat. All plots were given a basal application of 120 kg P ha^−1^ and 120 kg K ha^−1^. Nitrogen was supplied as urea (46.4% N), and P and K were applied as calcium superphosphate (12% P_2_O_5_) and potassium chloride (60% K_2_O), respectively. Each plot was 12 m^2^ (4 m × 3 m) with 50 cm row spacing between neighboring plots. Wheat cultivar ‘Ningmai 13’ (the use and collection of variety comply with national and international regulations) was sown on 5 November with row spacing of 20 cm and planting density of 300 × 10^4^ ha^−1^ for basic seedlings and was harvested on 25 May of the next year. The planting pattern of the experimental plot was wheat–rice rotation.

### 4.2. Sampling and Measurements

Leaf area of photosynthetic efficiency: the areas of the flag leaf, the second leaf, and the third leaf from the top of wheat plants were measured by a portable leaf area meter (Model Li-3000C, USA). Ten wheat plants were sampled from each treatment at middle of filling stage in 2020 and 2021, respectively, and the measurements were repeated ten times for each parameter.

Chlorophyll content: chlorophyll content was measured using a hand-held chlorophyll meter (*SPAD*-502, manufactured by the Konica Minolta Company, Tokyo, Japan, and measuring area was 2 mm × 3 mm); the same parts of flag leaves of the wheat plants were selected and measured at jointing, booting, and middle of filling stages in 2020 and 2021, respectively. Ten flag leaves in each treatment were sampled for measurement (each leaf was measured three times).

Canopy photosynthetically active radiation: photosynthetically active radiation (PAR) was measured by SUNSCAN Canopy Analysis System (Delta company, Britain) at flowering and middle of filling stages of wheat, and the photosynthetically active radiation of the canopy was calculated by the difference value between the photosynthetically active radiation of the top (approximately 1.5 m above ground level) and bottom (the transmitted PAR measured at the base of the canopy) of wheat canopy; the measurement time was 9:00–11:30 in the morning and 13:00–16:00 in the afternoon in 2020 and 2021, respectively. The spot measurements were limited to clear days to avoid poor quality of incident PAR (e.g., multiple sources of PAR due to cloud refraction and reflection), which influences the light interception measurements. Five measurements were taken for the ‘top’ and ‘bottom’ canopy position at locations selected randomly in each plot in rapid succession. 

Photosynthetic characteristics: leaf photosynthetic rate, stomatal conductance, transpiration rate, and intercellular CO_2_ concentration were measured with a portable photosynthesis system (LI-6400, Li-Cor, Lincoln, NE, USA) at 9:30–11:30 h local time at the flowering stage in 2020 and 2021. The flag leaf of wheat plant (five flag leaves in each plot) was selected for the leaf measurements.

Grain quality: protein (dry basis), starch, wet gluten (dry basis), kernel hardness, and sedimentation value in more than 1000 g of the intact seeds were measured by near-infrared spectroscopy with a Foss Infratec 1241 Grain Analyzer (Hillerød, Denmark). Each treatment was repeated three times, and the data was the average value of 2019, 2020, and 2021. 

Harvest index (HI) = grain yield/total above-ground biomass at physiological maturity.

### 4.3. Statistical Analysis

ANOVA was performed by using SPSS 20.0 software with the general linear model-univariate procedure (IBM, Armonk, New York, NY, USA). ANOVAs were performed with the N level and ratio of basal to topdressing as the main effects and included their interactions. All treatment means were compared for any significant differences by LSD multiple range tests at a significance level of *p* = 0.05 using the SPSS 20.0 software package for Windows [32]. 

## 5. Conclusions

The effects of N topdressing ratio on improving canopy photosynthesis and yield were consistently 6:4 > 5:5 > 7:3. N topdressing ratio 5:5 was more conducive to increasing grain protein content, wet gluten, kernel hardness, and sedimentation value. The effect of increasing N application rate was more considerable than that of N topdressing ratio. Increasing N by 120 kg ha^−1^ significantly improved photosynthetic efficiency leaf area of canopy, photosynthetic rate and grain quality, and increasing N by 60 kg ha^−1^ significantly enhanced canopy photosynthetically active radiation and yield. N3R2 (240 kg N ha^−1^ with N topdressing ratio 6:4) was more conducive to improving canopy photosynthesis, yield, and harvest index.

## Figures and Tables

**Table 1:** Effects of nitrogen application method on wheat leaf area of photosynthetic efficiency at middle of filling stage.

Nitrogen Fertilization	Ratio of Basal to Topdressing	2020			2021		
		Flag Leaf (cm^2^)	Second Leaf from Top (cm^2^)	Third Leaf from Top (cm^2^)	Flag Leaf (cm^2^)	Second Leaf from Top (cm^2^)	Third Leaf from Top (cm^2^)
N0	R0	12.30 ± 1.08 f	16.00 ± 0.79 f	18.83 ± 0.32 g	13.03 ± 0.40 f	16.43 ± 1.27 e	19.27 ± 0.49 f
N1	R1	14.17 ± 1.34 e	19.23 ± 0.60 e	25.27 ± 1.00 f	15.27 ± 0.91 e	20.07 ± 0.25 d	26.00 ± 0.36 e
	R2	15.33 ± 0.84 cde	20.10 ± 0.85 de	26.20 ± 0.56 def	15.57 ± 0.57 de	21.17 ± 1.07 bcd	27.07 ± 0.67 cde
	R3	14.80 ± 0.78 de	19.57 ± 0.95 de	25.77 ± 1.01 ef	15.47 ± 0.70 de	20.67 ± 0.96 cd	26.63 ± 0.85 de
N2	R1	15.93 ± 1.06 bcd	20.53 ± 0.70 cde	26.40 ± 0.95 cdef	16.10 ± 0.75 de	21.53 ± 0.71 abc	27.73 ± 0.60 bcd
	R2	17.03 ± 0.98 ab	21.77 ± 0.29 abc	27.27 ± 0.42 abcd	16.90 ± 0.87 bcd	22.17 ± 1.07 ab	28.63 ± 0.61 ab
	R3	16.50 ± 0.98 bc	20.97 ± 1.17 bcd	26.97 ± 0.40 bcde	16.50 ± 0.98 cde	21.77 ± 1.11 abc	28.40 ± 0.75 abc
N3	R1	17.87 ± 0.91 a	21.83 ± 0.75 abc	27.73 ± 0.71 abc	17.60 ± 1.18 abc	22.40 ± 0.98 ab	28.87 ± 1.05 ab
	R2	18.33 ± 0.67 a	22.70 ± 1.04 a	28.50 ± 1.00 a	18.43 ± 0.97 a	22.97 ± 1.27 a	29.30 ± 0.79 a
	R3	17.97 ± 0.86 a	22.40 ± 0.95 ab	28.20 ± 0.66 ab	18.23 ± 0.65 ab	22.63 ± 0.96 a	29.10 ± 1.30 ab

Note: values were means ± SD. Means followed by different letters in the same column indicate a significant difference (*p <* 0.05). Four nitrogen fertilization levels: N0, 0 kg ha^−1^; N1, 120 kg ha^−1^; N2, 180 kg ha^−1^; N3, 240 kg ha^−1^. Four ratios of basal N to topdressing: R0, 0:0; R1, 7:3; R2, 6:4; R3, 5:5.

**Table 2:** Effects of nitrogen application method on photosynthetic characteristics of flag leaf at flowering stage.

N Fertilization	Ratio of Basal to Topdressing	Photosynthetic Rate (µmol m^−2^s^−1^)	Stomatal Conductance (mol m^−2^s^−1^)	Transpiration Rate (mmol m^−2^s^−1^)	Intercellular CO_2_ Concentration (µmol mol^−1^)
2020					
N0	R0	8.27 ± 0.35 f	0.213 ± 0.02 f	2.50 ± 0.16 e	530.27 ± 14.46 a
N1	R1	14.33 ± 0.64 e	0.398 ± 0.03 de	3.84 ± 0.27 d	361.73 ± 18.07 b
	R2	14.57 ± 1.23 de	0.430 ± 0.03 cd	3.96 ± 0.20 cd	345.23 ± 9.92 bc
	R3	14.50 ± 0.66 e	0.417 ± 0.04 d	3.90 ± 0.20 cd	351.97 ± 25.07 bc
N2	R1	15.27 ± 0.95 cde	0.443 ± 0.04 bcd	3.99 ± 0.23 bcd	342.73 ± 13.90 bcd
	R2	16.10 ± 0.62 abc	0.487 ± 0.03 abc	4.23 ± 0.12 abc	324.87 ± 14.80 cde
	R3	15.87 ± 0.97 bcd	0.467 ± 0.03 abcd	4.13 ± 0.21 abcd	339.43 ± 12.95 bcd
N3	R1	16.57 ± 0.61 abc	0.497 ± 0.02 ab	4.19 ± 0.18 abcd	315.27 ± 18.77 de
	R2	17.30 ± 0.56 a	0.527 ± 0.06 a	4.41 ± 0.33 a	300.10 ± 19.03 e
	R3	17.13 ± 0.35 ab	0.503 ± 0.05 ab	4.35 ± 0.25 ab	306.43 ± 22.88 e
2021					
N0	R0	6.83 ± 0.35 f	0.197 ± 0.05 d	2.12 ± 0.14 d	589.60 ± 11.59 a
N1	R1	11.30 ± 0.80 e	0.327 ± 0.03 c	3.43 ± 0.14 c	405.47 ± 18.38 b
	R2	12.33 ± 0.55 de	0.360 ± 0.04 bc	3.56 ± 0.19 bc	387.80 ± 11.70 bc
	R3	11.67 ± 0.78 e	0.330 ± 0.01 c	3.49 ± 0.16 c	393.10 ± 22.81 bc
N2	R1	13.10 ± 0.70 cd	0.371 ± 0.05 bc	3.63 ± 0.23 bc	381.63 ± 16.05 cd
	R2	14.27 ± 0.59 abc	0.403 ± 0.05 ab	3.77 ± 0.23 abc	363.33 ± 13.18 def
	R3	13.73 ± 0.90 bc	0.390 ± 0.06 abc	3.70 ± 0.27 abc	379.27 ± 15.76 cde
N3	R1	14.93 ± 1.02 ab	0.413 ± 0.03 ab	3.86 ± 0.28 ab	358.27 ± 9.71 ef
	R2	15.33 ± 0.93 a	0.460 ± 0.04 a	4.03 ± 0.19 a	342.20 ± 16.98 f
	R3	15.10 ± 0.26 a	0.447 ± 0.04 a	3.99 ± 0.19 a	346.87 ± 9.31 f

Note: values were means ± SD. Means followed by different letters in the same column indicate a significant difference (*p <* 0.05). Four nitrogen fertilization levels: N0, 0 kg ha^−1^; N1, 120 kg ha^−1^; N2, 180 kg ha^−1^; N3, 240 kg ha^−1^. Four ratios of basal N to topdressing: R0, 0:0; R1, 7:3; R2, 6:4; R3, 5:5.

**Table 3:** Effects of nitrogen application method on grain quality (average value of 2019, 2020 and 2021) of wheat.

N Fertilization	Ratio of Basal to Topdressing	Protein (%)	Starch (%)	Wet Gluten Content (%)	Kernel Hardness	Sedimentation Value (mL)
N0	R0	11.27 ± 0.15 e	73.50 ± 1.25 a	22.47 ± 0.42 g	44.33 ± 1.06 c	22.80 ± 0.62 d
N1	R1	11.50 ± 0.36 de	69.43 ± 1.05 b	23.13 ± 0.76 fg	47.60 ± 0.82 ab	26.50 ± 0.90 c
	R2	11.53 ± 0.35 de	69.37 ± 0.67 b	23.23 ± 0.78 fg	47.23 ± 1.19 b	26.87 ± 0.31 c
	R3	11.63 ± 0.23 cde	69.03 ± 0.68 bc	23.53 ± 0.57 efg	47.50 ± 0.75 ab	27.13 ± 0.59 c
N2	R1	12.10 ± 0.44 bcd	67.97 ± 0.47 bcd	24.33 ± 0.64 def	48.37 ± 1.08 ab	29.20 ± 0.46 b
	R2	12.30 ± 0.66 abc	67.60 ± 0.26 cd	24.70 ± 0.60 cde	48.53 ± 1.00 ab	29.93 ± 1.21 b
	R3	12.43 ± 0.64 ab	67.50 ± 1.11 cd	24.80 ± 0.62 bcd	48.33 ± 1.36 ab	30.03 ± 0.67 b
N3	R1	12.60 ± 0.44 ab	66.93 ± 1.17 d	25.63 ± 1.05 abc	49.03 ± 0.50 a	31.43 ± 0.97 a
	R2	12.67 ± 0.40 ab	66.73 ± 1.16 d	25.97 ± 1.04 ab	49.07 ± 0.42 a	31.50 ± 0.90 a
	R3	12.83 ± 0.55 a	66.50 ± 1.25 d	26.17 ± 0.71 a	49.23 ± 1.03 a	32.03 ± 0.60 a

Note: values were means (2019, 2020 and 2021) ± SD. Means followed by different letters in the same column indicate a significant difference (*p <* 0.05). Four nitrogen fertilization levels: N0, 0 kg ha^−1^; N1, 120 kg ha^−1^; N2, 180 kg ha^−1^; N3, 240 kg ha^−1^. Four ratios of basal N to topdressing: R0, 0:0; R1, 7:3; R2, 6:4; R3, 5:5.

## Data Availability

Data is contained within the article.
